# Supplementary material for: Results of a Randomized, Double-Blind, Placebo-Controlled, Phase 1b/2 Trial of Nabpaclitaxel + Gemcitabine ± Olaratumab in Treatment-Naïve Participants with Metastatic Pancreatic Cancer
Source: Cancers (Basel). 2024 Mar 28;16(7):1323. doi: 10.3390/cancers16071323 (PMC11010910; doi:10.3390/cancers16071323)
Supplement: Supplementary file 1 [file cancers-16-01323-s001.zip › cancers-2841981-supplementary.pdf]

cancers

MDPI

Supplementary Materials:

Table S1. Phase 1: Demographic and baseline characteristics

Parameter	Cohort 1 Olaratumab 15 mg/kg plus NabPac Gem (N=3)	Cohort 2 Olaratumab 20 mg/kg plus NabPac Gem (N=7)	Cohort Expansion Olaratumab 20 mg/kg plus NabPac Gem (N=12)	Total (N=22) n (%)
**Age, years**	73.7±3.8	66.1±6.6	67.1±10.2	67.7±8.6
**Gender, Male**	1 (33.3)	4 (57.1)	9 (75.0)	14 (63.6)
**Race, White**	3 (100.0)	7 (100.0)	12 (100.0)	22 (100.0)
**BMI (kg/m²)**	29.9±5.8	27.7± 7.0	27.2 ±6.7	27.8 ±6.4
**Country, USA**	1 (33.3)	5 (71.4)	7 (58.3)	13 (59.1)
**Primary tumor present, yes**	3 (100.0)	6 (85.7)	10 (83.3)	19 (86.4)
**ECOG performance status**				
**0**	2 (66.7)	4 (57.1)	3 (25.0)	9 (40.9)
**1**	1 (33.3)	3 (42.9)	9 (75.0)	13 (59.1)
**2-5**	0	0	0	0

Abbreviations: BMI, body mass index; ECOG, Eastern Cooperative Oncology Group; Gem, gemcitabine; N, number of subjects in Safety Population; n, number of subjects within category; NabPac, nabpaclitaxel; SD, standard deviation.
Data are mean±SD or n (%); Data cut-off date = 30 July 2018.

Table S2. Serious adverse events reported in Phase 1b and Phase 2 of the study

Phase 1b

Preferred term, n (%)	Cohort 1 Olaratumab 15 mg/kg plus NabPac Gem (N=3)	Cohort 2 Olaratumab 20 mg/kg plus NabPac Gem (N=7)	Cohort Expansion Olaratumab 20 mg/kg plus NabPac Gem (N=12)	Total (N=22)
Subjects with ≥1 SAE	2 (66.7)	3 (42.9)	4 (33.3)	9 (40.9)
Nausea	0	0	1 (8.3)	1 (4.5)
Pancreatic cyst	1 (33.3)	0	0	1 (4.5)
Vomiting	0	0	1 (8.3)	1 (4.5)
Asthenia	0	0	1 (8.3)	1 (4.5)
Pyrexia	0	0	1 (8.3)	1 (4.5)
Cerebrovascular accident	0	0	1 (8.3)	1 (4.5)
Epilepsy	0	1 (14.3)	0	1 (4.5)
Septic shock	1 (33.3)	0	0	1 (4.5)
Infusion-related reactions	0	1 (14.3)	0	1 (4.5)
Neutrophil count decreased	1 (33.3)	0	0	1 (4.5)
Confusional state	1 (33.3)	0	0	1 (4.5)
Pneumonitis	0	1 (14.3)	0	1 (4.5)

Phase 2

	Olaratumab arm (N=81)		Placebo arm (N=78)		Total (N=159)	
	Any grade n (%)	Grade 3/4/5 n (%)	Any grade n (%)	Grade 3/4/5 n (%)	Any grade n (%)	Grade 3/4/5 n (%)
Subjects with ≥1 related SAE	18 (22.2)	14 (17.3)	10 (12.8)	9 (11.5)	28 (17.6)	23 (14.5)
Febrile neutropenia	0	0	1 (1.3)	1 (1.3)	1 (0.6)	1 (0.6)
Cardiac arrest	1 (1.2)	1 (1.2)	0	0	1 (0.6)	1 (0.6)
Cardiac failure congestive	0	0	1 (1.3)	1 (1.3)	1 (0.6)	1 (0.6)

Diarrhea	2 (2.5)	1 (1.2)	2 (2.6)	2 (2.6)	4 (2.5)	3 (1.9)
Vomiting	2 (2.5)	2 (2.5)	1 (1.3)	1 (1.3)	3 (1.9)	3 (1.9)
Nausea	1 (1.2)	1 (1.2)	1 (1.3)	1 (1.3)	2 (1.3)	2 (1.3)
Intestinal perforation	1 (1.2)	1 (1.2)	0	0	1 (0.6)	1 (0.6)
Colitis	0	0	1 (1.3)	1 (1.3)	1 (0.6)	1 (0.6)
Pyrexia	4 (4.9)	0	1 (1.3)	0	5 (3.1)	0
Asthenia	1 (1.2)	1 (1.2)	0	0	1 (0.6)	1 (0.6)
Chills	1 (1.2)	0	0	0	1 (0.6)	0
Fatigue	1 (1.2)	0	0	0	1 (0.6)	0
Sepsis	2 (2.5)	2 (2.5)	0	0	2 (1.3)	2 (1.3)
Pneumonia	1 (1.2)	1 (1.2)	1 (1.3)	1 (1.3)	2 (1.3)	2 (1.3)
Gastroenteritis	1 (1.2)	1 (1.2)	0	0	1 (0.6)	1 (0.6)
Liver abscess	1 (1.2)	1 (1.2)	0	0	1 (0.6)	1 (0.6)
Pneumocystis jirovecii pneumonia	1 (1.2)	1 (1.2)	0	0	1 (0.6)	1 (0.6)
Clostridium difficile colitis	0	0	1 (1.3)	1 (1.3)	1 (0.6)	1 (0.6)
Pneumonia Klebsiella	0	0	1 (1.3)	1 (1.3)	1 (0.6)	1 (0.6)
Platelet count decreased	1 (1.2)	1 (1.2)	0	0	1 (0.6)	1 (0.6)
Hyponatremia	1 (1.2)	0	0	0	1 (0.6)	0
Myositis	0	0	1 (1.3)	1 (1.3)	1 (0.6)	1 (0.6)
Ischemic cerebral infarction	1 (1.2)	1 (1.2)	0	0	1 (0.6)	1 (0.6)
Memory impairment	1 (1.2)	1 (1.2)	0	0	1 (0.6)	1 (0.6)
Seizure	1 (1.2)	1 (1.2)	0	0	1 (0.6)	1 (0.6)
Leuko-encephalopathy	0	0	1 (1.3)	1 (1.3)	1 (0.6)	1 (0.6)
Dyspnea	1 (1.2)	1 (1.2)	0	0	1 (0.6)	1 (0.6)

Respiratory failure	1 (1.2)	1 (1.2)	0	0	1 (0.6)	1 (0.6)
Pleural effusion	0	0	1 (1.3)	1 (1.3)	1 (0.6)	1 (0.6)
Hypotension	0	0	1 (1.3)	1 (1.3)	1 (0.6)	1 (0.6)

Abbreviations: CTCAE, Common Terminology Criteria for Adverse Events; Gem, gemcitabine; N, number of subjects in safety population; n, number of subjects within category; NabPac, nabpaclitaxel; SAE, serious adverse event.
SAEs presented are by maximum CTACE grade categories preferred term by decreasing frequency within system organ class.

Table S3. Comparison of olaratumab pharmacokinetic parameter estimates from the previous population PK analysis vs updated population PK analysis

Parameter Description	Previous Analysis Population Estimate (%SEE)	Previous Analysis Inter-Patient Variability (%SEE)	Current Analysis Population Estimate (%SEE)	Current Analysis Inter-Patient Variability %CV (%SEE)
**Structural Model**				
Clearance, CL (L/h)	0.0186 (1.4)	34.6 (9.8)	0.0191 (2.3)	36.5 (15.4)
Central Volume of Distribution, V_1 (L)	3.43 (1.1)	26.2 (14)	3.47 (1.1)	28.1 (14.1)
Peripheral Volume of Distribution, V_2 (L)	1.62 (9.4)	-	1.91 (7.8)	-
Inter-compartmental clearance rate, Q (L/h)	0.0458 (15.0)	-	0.0412 (20.7)	-
**Covariate Effects**				
WTE_{CL}[a]	0.518 (9.1)	-	0.465 (18.4)	-
WTE_{V1}[b]	0.612 (7.7)	-	0.624 (6.7)	-
$TUMR_{CL}$[a]	0.00106 (15.4)		0.00106 (26.9)	
**Residual Error**				
Additive (µg/mL)	25 (25.6)		11.7 (26.8)	
Proportional	24.7 (5.5)		27.7 (3.9)	

Abbreviations: CV, coefficient of variation; PK, pharmacokinetic; POP PK, population PK; SEE, standard error of the estimate; TUMRCL, tumor size effect on clearance; WTECL, body weight effect on clearance; WTEV1, body weight effect on central volume of distribution.

[a]CLind = CL * ((WTE/76.0)^WTECL) * (1 + TUMRCL * (TUMR-89))

[b]V1ind = V1 * ((WTE/76.0)^WTEV1)

Note: Median data from previous POP PK models used. Weight = 76.0 kg and Tumor size = 89 mm.
